# Factors Associated with Increased Risk of Early Severe Neonatal Morbidity in Late Preterm and Early Term Infants

**DOI:** 10.3390/jcm10061319

**Published:** 2021-03-23

**Authors:** Tesfaye S. Mengistu, Veronika Schreiber, Christopher Flatley, Jane Fox, Sailesh Kumar

**Affiliations:** 1 Mater Research Institute, University of Queensland, Level 3 Aubigny Place, Raymond Terrace, South Brisbane, QLD 4101, Australia; t.mengistu@uq.net.au (T.S.M.); veronika.schreiber@mater.uq.edu.au (V.S.); cjflatley@gmail.com (C.F.); jane.fox@mater.uq.edu.au (J.F.); 2 School of Public Health, College of Medicine and Health Sciences, Bahir Dar University, P.O. Box 79, Bahir Dar 6000, Ethiopia; 3 Faculty of Medicine, The University of Queensland, Herston, QLD 4006, Australia; 4 NHMRC Center of Research Excellence in Stillbirth, Mater Research Institute, University of Queensland, Raymond Terrace, South Brisbane, QLD 4101, Australia

**Keywords:** late preterm, early term, risk factor, severe neonatal morbidity

## Abstract

Although the risk of neonatal mortality is generally low for late preterm and early term infants, they are still significantly predisposed to severe neonatal morbidity (SNM) despite being born at relatively advanced gestations. In this study, we investigated maternal and intrapartum risk factors for early SNM in late preterm and early term infants. This was a retrospective cohort study of non-anomalous, singleton infants (34^+0^–38^+6^ gestational weeks) born at the Mater Mother’s Hospital in Brisbane, Australia from January 2015 to May 2020. Early SNM was defined as a composite of any of the following severe neonatal outcome indicators: admission to neonatal intensive care unit (NICU) in conjunction with an Apgar score <4 at 5 min, severe respiratory distress, severe neonatal acidosis (cord pH < 7.0 or base excess <−12 mmol/L). Multivariable binomial logistic regression analyses using generalized estimating equations (GEE) were used to identify risk factors. Of the total infants born at 34^+0^–38^+6^ gestational weeks, 5.7% had at least one component of the composite outcome. For late preterm infants, pre-existing diabetes mellitus, instrumental birth and emergency caesarean birth for non-reassuring fetal status were associated with increased odds for early SNM, whilst for early term infants, pre-existing and gestational diabetes mellitus, antepartum hemorrhage, instrumental, emergency caesarean and elective caesarean birth were significant risk factors. In conclusion, we identified several risk factors contributing to early SNM in late preterm and early term cohort. Our results suggest that predicted probability of early SNM decreased as gestation increased.

## 1. Introduction

The causes of severe neonatal morbidity (SNM) are multifactorial, although gestational age at birth is a major contributor to its development [1,2,3]. Whilst advances in obstetric, intrapartum and neonatal care have resulted in significant improvements in birth outcomes particularly a reduction in neonatal mortality, prevention of SNM remains challenging. Although it is known that SNM is associated with higher risk of perinatal death [1,2,3], some cohorts of infants are particularly vulnerable—those that are preterm, growth restricted or small or large for gestational age and those with hypoxic intrapartum events requiring operative birth. Maternal factors also appear to play a role in potentiating severe neonatal outcomes.

Late preterm (34^+0^–36^+6^ gestational weeks) and early term (37^+0^–38^+6^ gestational weeks) infants are at increased risk for morbidity and mortality compared to those born at term, [4,5,6,7] albeit with significantly lower risk than that of infants born < 32 gestational weeks [8,9]. Overall, preterm birth rates even in high-income countries (HIC) [2,3] continue to rise and it remains a very strong predictor of neonatal morbidity [3,10]. Late preterm birth in particular is a major contributor [11,12] accounting for up to 72% of all preterm births [13,14].

SNM continues to impose a significant burden on healthcare resources—these infants often require prolonged neonatal support and longer overall hospital stays. It is therefore important to understand the antecedents of severe neonatal morbidity in the late preterm and/or early term cohort as this may stimulate research and provide better understanding of preventative and therapeutic measures to minimize this healthcare burden. The aim of this study, therefore, was to identify risk factors for SNM in late preterm and early term infants.

## 2. Materials and Methods

This was a retrospective cohort study of non-anomalous, late preterm, and early term singleton infants born at the Mater Mother’s Hospital in Brisbane, Australia between January 2015 and May 2020.

Gestational age was estimated using the last menstrual period or earliest ultrasound measurements. Although early ultrasound is a gold standard estimation technique, last menstrual period is also a reliable estimation technique in early gestational weeks. Early severe neonatal morbidity (SNM) was defined as the presence of at least one of the following elements: Apgar score < 4 at 5 min or severe respiratory distress requiring respiratory support or severe neonatal acidosis (cord artery pH < 7.0 or base excess <−12 mmol/L) and admission to the neonatal intensive care unit (NICU).

Maternal demographic and obstetric variables, including age at birth, ethnicity (Caucasian, Asian, Indigenous, or other), socio-economic index for areas (SEIFA) score, smoking, use of illicit drugs during pregnancy, nulliparity, body mass index (BMI), antepartum hemorrhage, chorioamnionitis, diabetes mellitus [pre-existing (Type 1 and Type 2) and gestational] and hypertension (chronic, gestational and pre-eclampsia), and use of assisted reproduction technology (ART) were extracted from the institution’s perinatal database. Intrapartum variables included onset of labour [spontaneous or induction of labour (IOL)], mode of birth (spontaneous vaginal delivery (SVD), instrumental, caesarean section (CS)) and indication (non-reassuring fetal status (NRFS) or failure to progress (FTP) or other) for emergency caesarean section.

Ethnicity, smoking and illicit drug use during pregnancy were self-reported variables. The SEIFA index is an Australian area-based socio-economic score which is reflective of socio-economic status [15]. A score in the lowest quintile is indicative of significant socio-economic disparity. Chorioamnionitis was a clinical diagnosis made by the treating intrapartum obstetric team usually on the basis of offensive liquor and maternal pyrexia.

### Statistical Analysis

Data measured on a continuous scale are reported as mean (standard deviation) or median (interquartile range) and differences were analyzed using the Mann-Whitney U-test (Wilcoxon rank-sum test). Proportions are reported as numbers of observations and percentage, differences were analyzed using the chi-squared test. Pre-pregnancy body mass index (BMI) (kg/m^2^) and infant birthweight were used as continuous covariates.

Model building was performed stratified by gestational age categories (late preterm vs. early term). Sensitivity analyses were undertaken to assess the impact of missed observations of any particular variable on the outcome. Binomial logistic regression analyses using the generalized estimating equation (GEE) with exchangeable correlation and robust variance estimation technique was used throughout the analysis to account for women birthing multiple times over the study period. A purposeful model building approach as described by Hosmer and Lemeshow [16] was used to identify and include clinically important risk factors for early SNM. Risk factors that were significantly associated with early SNM (*p* ≤ 0.2) [16] in the univariable regression analysis were considered in the multivariable models at *p* < 0.05 criterion. The impact of gestational age on the association of risk factors and the outcome variable was assessed by adjusting each univariable regression for gestational age at birth. We used the backward elimination technique guided by a combination of low correlation between predictor variables [17] and event per variable (EPV ≥ 10) as previously described [18,19]. We also performed sensitivity analysis if the numbers of missing observations for certain variable were increased.

All multivariable models were assessed for confounding and potential interactions between risk factors and their overall compliance with model assumptions. Multivariable models were compared using quasi-likelihood information criteria (QIC) [20] to identify the most parsimonious multivariable regression model. Given that gestational age is significantly correlated with several adverse neonatal outcomes, [21] post-estimation marginal probabilities of early SNM by gestational age was assessed for the entire cohort.

The STATA Statistical Software: Release 16 (StataCorp. 2019, StataCorp LLC, College Station, TX, USA) was used for data analyses. A *p*-value < 0.05 was considered significant.

## 3. Results

**Figure 1:**
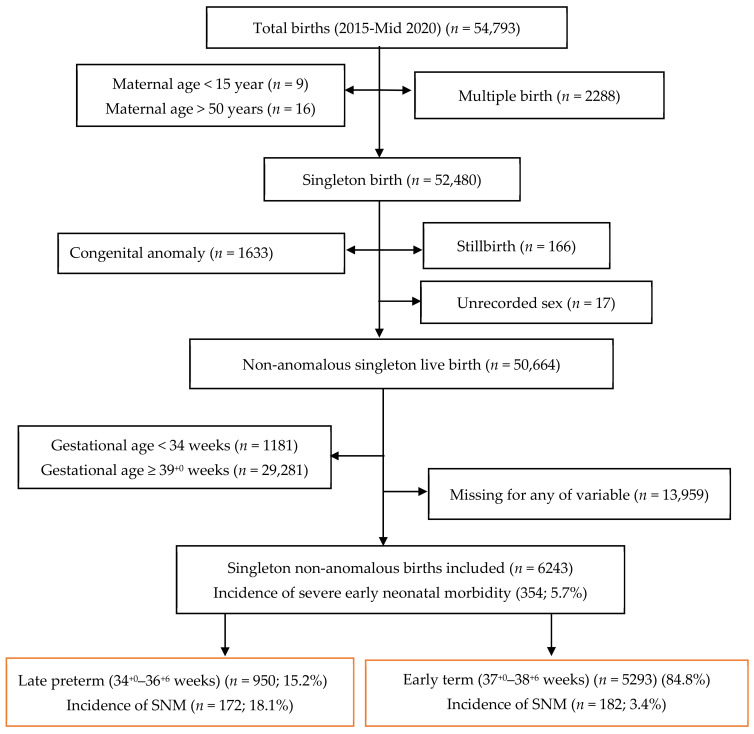
Participant selection flow diagram.

Selection of the study cohorts is presented in Figure 1. After excluding: infants born to women aged ≤ 15 or > 50 years (*n* = 25), multiple births (*n* = 2288), major congenital malformations (*n* = 1633), stillbirths (*n* = 166), infants with unrecorded sex (*n* = 17), early preterm (< 34 gestational weeks) (*n* = 1181) or full term, late and post-term (≥ 39^+0^ gestational weeks) gestation (*n* = 29,281) and those with missing values for any of the relevant predictors (*n* = 13,959), the final study cohort comprised of 6243 infants. Of these, the late preterm cohort consisted of 950 (15.2%) infants whilst the early term group included 5293 (84.8%) infants.

The overall prevalence of early SNM was 5.7% (354/6243) with a higher proportion seen in late preterm (172/950, 18.1%) compared to early term (182/5293, 3.4%) infants (Figure 1). Table 1 summarizes demographic, antepartum, intrapartum and neonatal risk factors for SNM for both cohorts.

To assess the influence of gestational age at birth on early SNM in both cohorts, multivariable analyses adjusting only for gestational age was performed (Table 2). In the late preterm cohort, pre-existing diabetes (OR 3.27, 95%CI: 1.62–6.63), elective CS (OR 1.63, 95%CI: 1.00–2.70) and emergency CS for NRFS (OR 3.20, 95%CI: 1.53–6.69) were associated with increased odds of early SNM. Conversely, nulliparity (OR 0.59, 95%CI: 0.42–0.83), Asian ethnicity (OR 0.59, 95%CI: 0.37–0.96) and female sex (OR 0.59, 95%CI: 0.42–0.84) resulted in lower odds of SNM.

In the early term cohort, nulliparity (OR 1.38, 95%CI: 1.03–1.86), raised maternal BMI (OR 1.03, 95%CI: 1.01–1.05), pre-existing diabetes (OR 4.20, 95%CI: 2.48–7.09), gestational diabetes (OR 1.45, 95%CI: 1.06–1.99), instrumental birth (OR 3.46, 95%CI: 2.18–5.49), elective CS (OR 1.72, 95%CI: 1.13–2.60), and emergency CS for FTP (OR 3.17, 95%CI: 1.63–6.17) or NRFS (OR 3.17, 95%CI: 1.59–6.33) were associated with increased odds of early SNM. Similar to the late preterm cohort, Asian ethnicity and female sex were also associated with lower odds of early SNM.

**Figure 2:**
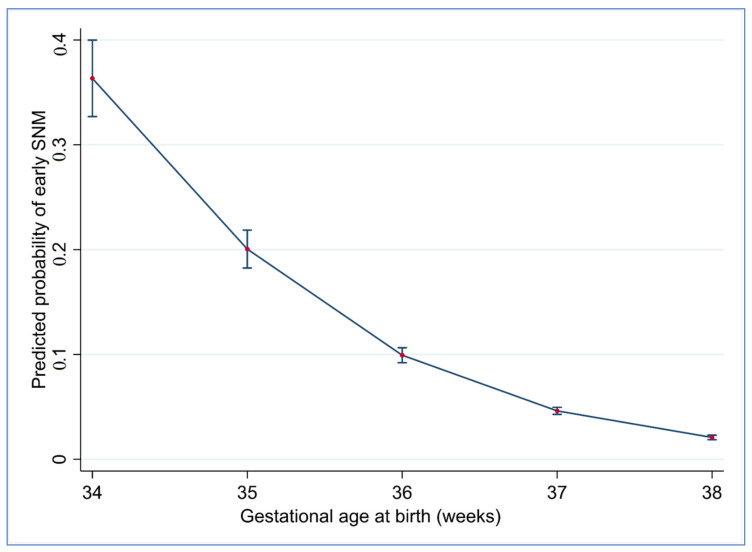
Adjusted predicted probability for early SNM by gestational week.

**Figure 3:**
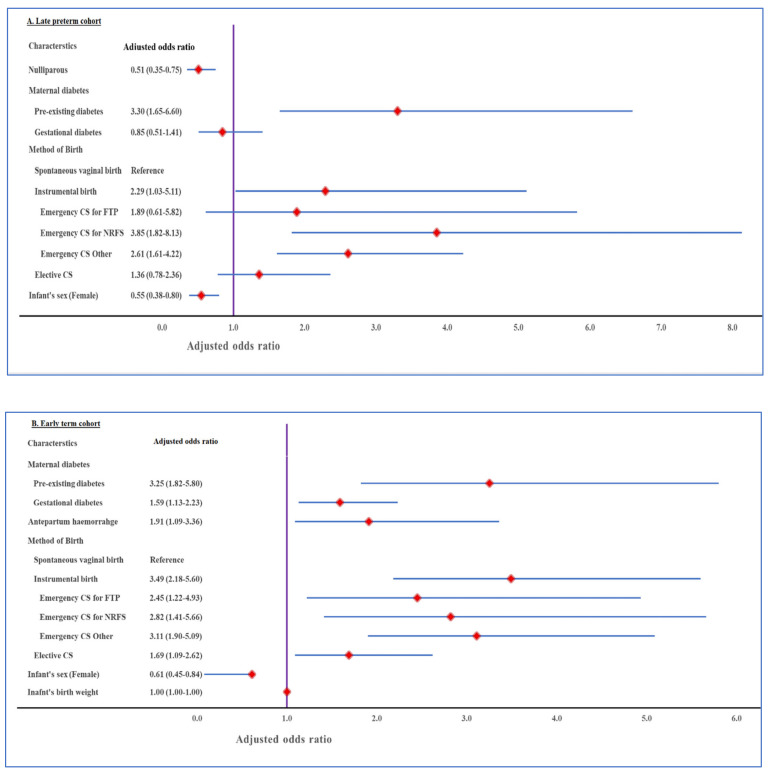
Adjusted associations of risk factors with early severe neonatal morbidity (SNM) in late preterm (**A**) and early term (**B**) infants. A—adjusted for: maternal age, ethnicity, gestational age at birth in weeks and B—adjusted for maternal age, ethnicity, maternal body mass index, SEIFA Score and gestational age at birth.

In the final multivariable analysis, the predicted probability of early SNM significantly decreased as gestation increased (Figure 2). After adjusting for all confounders (maternal age, ethnicity, maternal BMI, SEIFA Score and gestational age at birth) identified by univariable analysis, we found that pre-existing diabetes, instrumental birth and emergency CS for NRFS were associated with higher odds of early SNM in late preterm infants. Nulliparity and female sex remained associated with lower odds of this outcome. For early term infants, pre-existing and gestational diabetes mellitus, antepartum hemorrhage, instrumental birth, emergency CS for NRFS, FTP or other indications and elective CS were all associated with higher odds of early SNM. Conversely, higher birthweight and female sex were associated with lower odds of early SNM (Figure 3 and Supplementary Materials Table S1).

NB: Analysis adjusted for: maternal age, ethnicity, gestational age at birth, maternal body mass index and SEIFA Score.

During our descriptive analysis, we observed higher numbers of missing observations for the antepartum hemorrhage variable (Supplementary Materials Table S2). However, sensitivity analysis confirmed the associations between risk factors and SNM were not influenced by the inclusion of women with missed observations for antepartum hemorrhage in the final analysis (Supplementary Materials Tables S3 and S4).

## 4. Discussion

The results of this large single center, observational study identified several key risk factors for early SNM in late preterm and early term infants. Although both cohorts had some common risk factors (pre-existing diabetes mellitus and emergency CS for NRFS) we found that there was discordance for other risk factors. For example, nulliparity was only associated with lower odds of SNM in the late preterm cohort whilst antepartum hemorrhage was only associated with increased odds only in the early term cohort.

It has been reported that smoking [22,23] and illicit drug use during pregnancy [24,25] are associated with several adverse perinatal outcomes. In Australia, indigenous women have significantly poorer neonatal outcomes attributable to smoking, alcohol and substance misuse, and assault [26]. However, our finding did not show correlation between smoking or illicit drug use with early SNM in both cohorts.

Surprisingly we did not find associations between maternal obesity and hypertension, and neonatal morbidity. However, maternal obesity could affect adverse neonatal outcome through increasing the risk of diabetes mellitus, hypertension which also increases the risk of indicated operative births in late preterm or early term gestations [5]. In agreement with our study, some studies show that hypertension is not associated with composite adverse perinatal outcome [27]. Nevertheless, the reasons for this dichotomy are not clear from our analysis but the relationship between maternal and sociodemographic factors and serious neonatal morbidity is complex and often inter-related—a recent large study from the United States showed that younger, white, less educated, and publicly insured women with more medical comorbidities were more likely to experience serious neonatal morbidity [28].

Our data also demonstrates that the predicted probability for early SNM was greatest at 34^+0^ gestational weeks and lowest at 38^+6^ gestational weeks, suggesting that gestational maturity at birth is a major influence on neonatal outcomes. This is broadly similar to another large North American study which showed that the hig1hest rates of neonatal morbidity were seen at 34 weeks gestation and declined thereafter [1]. These findings reflect the fact that late preterm and early term infants are physiologically and metabolically less mature than late term infants and are thus more susceptible to complications after birth. Respiratory morbidity in particular is heavily dependent on gestation at birth [29]. Our results are also consistent with other studies demonstrating increasing rates of composite neonatal morbidity, respiratory distress and neonatal intensive care unit admission with decreasing gestational age [30,31]. Indeed, it is reported that almost 1 in 5 of late preterm infants admitted to a neonatal intensive care unit have significant respiratory compromise [32]. Late preterm infants are often delivered because of a medical indication—in one study from the United States > 75% of late preterm births were medically indicated or spontaneous [33] with 25–45% medically indicated late preterm births [34]. We were however unable to accurately determine precise reasons for induction of labour in our study cohort, although the vast majority would have been medically indicated. Interestingly, induction of labour rates were higher in the early term cohort, probably reflecting a lower threshold for recommending delivery in view of the relatively advanced gestational age.

Several other findings from our study warrant noting. Firstly, rates of uncomplicated vaginal birth of affected infants in both gestational cohorts were low. Secondly, emergency caesarean rates for NRFS were almost twice [35] that for infants at term at our institution and thirdly pre-existing diabetes mellitus was associated with a 3–4-fold increase in odds of early SNM. We have previously shown that the mode of birth can influence neonatal outcomes with instrumental and caesarean birth being significant risk factors [36,37].

In our study, 5.7% of infants born late preterm or early term had early SNM. However, the prevalence of SNM is heavily dependent on its definition [7]. Our results nevertheless are very consistent with other published data in larger population studies [38]. Indeed, a British study by Knight et al. [39] showed that in their cohort, 5.4% of live born infants had at least one component of a composite neonatal adverse outcome indicator and that this was associated with a significantly greater risk of mortality or overnight hospital readmission. Our findings of the increased predicted probability of early SNM in low birth weight infants is also consistent with other studies [21,40,41].

Recent studies have shown that countries with higher preterm birth rates also have higher early term birth [42]. This would suggest that the etiologic factors responsible for late preterm birth overlap with those for early term birth. Indeed, conditions like pre-eclampsia and late fetal growth restriction are common in the late third trimester and contribute to rates of neonatal morbidity [5]. Delnord and Zeitlin [5] in their review point out that the shared determinants to late preterm and early term birth can be mitigated through potentially modifiable population and healthcare factors. In Australia, Newnham et al. suggest a similar approach with careful analysis of risk factors and aggressive treatment of modifiable precipitants of preterm birth.

The use of clinically relevant variables strongly reflective of neonatal morbidity and which are known to impact long term health is a major strength of this study [43]. We have also used robust statistical techniques to take clustering of observations into account which is a common limiting factor in panel data analysis. We also adjusted and stratified outcomes by gestational age categories and adjusted for several clinical confounders. We, however, acknowledge a number of limitations which are inherent to all statistical analysis derived from retrospective data. Firstly, recall bias may have influenced the accuracy of self-reported variables such as smoking and illicit drug use. Additionally, changes in obstetric and neonatal practice over the study period may have influenced some of the outcomes. Furthermore, although we used a composite definition of SNM it is recognized that there is no “gold standard” for this definition and therefore the generalizability of our results may be variable [8]. Another consideration between our study and other publications is that our study reflects a more contemporary cohort albeit one from a tertiary center. Comparisons with older studies are problematic because wider use of antenatal maternal steroids and advances in neonatal medicine (for example, changes in ventilator strategies for respiratory complications) may result in lower rates of SNM in a more recent cohort. However, we are also aware that it is difficult to truly be certain what influence practice change would have had on clinical outcomes as change is often gradual and improvements in outcome incremental over time.

## 5. Conclusions

In this study, we identified important risk factors for early SNM in late preterm and early term infants. Our results may be useful to clinicians because, although one approach to improving perinatal outcomes is by reducing unnecessary iatrogenic late preterm and early term births [44,45], recognition and mitigation of the risk conferred by certain factors associated with SNM is also important as part of an overall strategy to improve clinical outcomes.

## Figures and Tables

**Table 1:** Maternal demographics, antepartum and intrapartum variables.

Variables	Late Preterm Cohort	Early Term Cohort
	Early SNM Present (*n* = 172)	Early SNM Present (*n* = 182)
Maternal age (mean, sd) year	32.3 (5.3)	32.0 (5.4)
Ethnicity		
Caucasian	68.6% (118/172)	67.0% (122/182)
Indigenous	2.9% (5/172)	2.7% (5/182)
Asian	15.7% (27/172)	19.2% (35/182)
Other	12.8% (22/172)	11.0% (20/182)
SEIFA Score (median, IQR)	1035 (999–1067)	1035 (996–1067)
Smoking		
No smoking	14.5% (25/172)	76.9% (140/182)
Mother smokes	7.6% (13/172)	12.1% (22/182)
Partner smokes	5.8% (10/172)	11.0% (20/182)
Illicit drug use	5.8% (10/172)	6.6% (12/182)
Nulliparity	40.1% (69/172)	51.6% (94/182)
Maternal BMI (kg/m^2^)	23.9 (20.4–28.2)	26.0 (22.0–31.6)
Maternal diabetes status		
No diabetes	70.9% (122/172)	48.4% (88/182)
Pre-existing diabetes	12.8% (22/172)	11.0% (20/182)
Gestational diabetes	16.3% (28/172)	40.7% (74/182)
Hypertension		
No hypertension	81.4% (140/172)	85.7% (156/182)
Essential/gestational hypertension	11.0% (19/172)	8.8% (16/182)
Pre-eclampsia/Eclampsia/HELLP syndrome	7.6% (13/172)	5.5% (10/182)
Assisted reproduction	13.4% (23/172)	10.4% (19/182)
Chorioamnionitis	4.7% (8/172)	0.0% (0/182)
Antepartum haemorrhage	15.7% (27/172)	8.2% (15/182)
Induction of labour (IOL)	15.1% (26/172)	46.7% (85/182)
Method of birth		
Spontaneous vaginal birth	22.7% (39/172)	22.5% (41/182)
Instrumental birth	6.4% (11/172)	19.2% (35/182)
Emergency CS for FTP	1.7% (3/172)	6.6% (12/182)
Emergency CS for NRFS	7.6% (13/172)	6.0% (11/182)
Emergency CS Other	42.4% (73/172)	17.6% (32/182)
Elective CS	19.2% (33/172)	28.0% (51/182)
Birth weight (g) (mean, sd)	2614 (577)	3343 (562)
Infant’s sex		
Male	62.2% (107/172)	65.4% (119/182)
Female	37.8% (65/172)	34.6% (63/182)

sd—standard deviation, IQR—inter quartile range; BMI—body mass index; HELLP—hemolysis, elevated liver enzymes, low platelet count; IOL—induction of labour; CS–caesarean section; FTP—failure to progress; NRFS—non-reassuring fetal status.

**Table 2:** Unadjusted and gestational week adjusted association of risk factors and early severe neonatal morbidity (SNM) in late preterm and early term infants.

Variables	Late Preterm Cohort (*n* = 950)		Early Term Cohort (*n* = 5293)	
	Unadjusted OR (95%CI)	Adjusted OR (95%CI) ^†^	Unadjusted OR (95%CI)	Adjusted OR (95%CI) ^†^
Maternal age (mean, sd) year	0.99 (0.96–1.03)	1.00 (0.97–1.03)	0.98 (0.95–1.00)	0.98 (0.95–1.01)
Ethnicity				
Caucasian	Reference	Reference	Reference	Reference
indigenous	0.75 (0.28–2.00)	0.72 (0.27–1.93)	0.57 (0.23–1.41)	0.53 (0.21–1.33)
Asian	0.66 (0.42–1.04)	0.59 (0.37–0.96) *	0.65 (0.44–0.95) *	0.65 (0.45–0.96) *
Other	0.87 (0.53–1.45)	0.84 (0.50–1.43)	0.76 (0.47–1.23)	0.76 (0.47–1.23)
SEIFA Score (median, IQR)	1.00 (0.00–1.00)	1.00 (1.00–1.00)	1.00 (1.00–1.00) *	1.00 (1.00–1.00)
Smoking				
No smoking	Reference	Reference	Reference	Reference
Mother smokes	0.90 (0.54–1.51)	0.77 (0.46-1.29)	1.36 (0.86–2.14)	1.26 (0.79–2.00)
Partner smokes	0.86 (0.45–1.68)	0.69 (0.36–1.33)	1.61 (0.99–2.60)	1.60 (0.99–2.60)
Illicit drug use	0.75 (0.38–1.48)	0.57 (0.29–1.14)	1.18 (0.65–2.15)	1.14 (0.62–2.09)
Nullipara	0.64 (0.46–0.89) **	0.59 (0.42–0.83) **	1.40 (1.04–1.88) *	1.38 (1.03–1.86) *
Maternal BMI (kg/m^2^)	0.99 (0.96–1.02)	0.98 (0.96–1.01)	1.03 (1.01–1.05) ***	1.03 (1.01–1.05) ***
Maternal diabetes Status				
No diabetes	Reference	Reference	Reference	Reference
Pre-existing diabetes	2.91 (1.51–5.60) ***	3.27 (1.62–6.63) ***	4.67 (2.79–7.82) ***	4.20 (2.48–7.09) ***
Gestational diabetes	0.72 (0.45–1.14)	0.75 (0.46–1.22)	1.39 (1.01–2.00) *	145 (1.06–1.99) *
Hypertension				
No Hypertension	Reference	Reference	Reference	Reference
Essential/gestational hypertension	1.01 (0.63–1.62)	0.97 (0.57–1.66)	1.33 (0.79–2.24)	1.19 (0.70–2.02)
Pre-eclampsia/Eclampsia/HELLP syndrome	0.70 (0.38–1.31)	0.73 (0.38–1.40)	1.59 (0.82–3.06)	1.22 (0.63–2.37)
Assisted reproduction	0.77 (0.45–1.34)	0.86 (0.50–1.47)	0.79 (0.49–1.28)	0.77 (0.47–1.24)
Chorioamnionitis	1.14 (0.52–2.53)	0.81 (0.36–1.85)	--	--
Antepartum hemorrhage	1.51 (0.95–2.42)	1.23 (0.75–2.01)	1.80 (1.05–3.11) *	1.64 (0.95–2.84)
Induction of labour (IOL)	0.64 (0.41–1.00) *	0.76 (0.48–1.20)	1.19 (0.88–1.60)	1.21 (0.90–1.62)
Method of birth				
Spontaneous vaginal birth	Reference	Reference	Reference	Reference
Instrumental birth	1.50 (0.74–3.04)	1.78 (0.84–3.80)	3.45 (2.17–5.46) ***	3.46 (2.18–5.49) ***
Emergency CS for FTP	1.16 (0.33–4.07)	1.53 (0.44–5.25)	3.09 (1.60–5.58) ***	3.17 (1.63–6.17) ***
Emergency CS for NRFS	2.99 (1.48–6.04) **	3.20 (1.53–6.69) **	3.23 (1.63–6.40) ***	3.17 (1.59–6.33) ***
Emergency CS Other	2.62 (1.71–3.99) ***	2.57 (1.67–3.98) ***	3.53 (2.20 -5.66) ***	3.21 (1.99–5.18) ***
Elective CS	1.65 (1.00–2.71) *	1.63 (0.99–2.70)	1.66 (1.10–2.52) *	1.72 (1.13–2.60) *
Birth weight (g) (mean, sd)	1.00 (1.00–1.00)	1.00 (1.00–1.00)	1.00 (1.00–1.00) **	1.00 (1.00–1.00) ***
Infant’s sex (Female vs. male)	0.60 (0.43–0.84) **	0.59 (0.42–0.84) **	0.58 (0.43–0.79) ***	0.58 (0.42–0.79) ***

sd—standard deviation, IQR—inter quartile range; BMI—Body mass index; HELLP—hemolysis, elevated liver enzymes, low platelet count; IOL—induction of labour; CS—caesarean section; FTP—failure to progress; NRFS—non-reassuring fetal status; **^†^** Adjusted only for gestational age in weeks, * *p* < 0.05, ** *p* < 0.01, *** *p* < 0.001.

## Data Availability

The data presented in this study are available in the article or Supplementary Materials.

## Supplementary Materials

The following are available online at https://www.mdpi.com/2077-0383/10/6/1319/s1, Table S1: Multivariable association of risk factors and early severe neonatal morbidity by gestational age category, Table S2: Sensitivity analysis: Outcome and risk factors between cohorts with antepartum hemorrhage complete observation and no complete data, Table S3: Sensitivity analysis of adjusted regression model for late preterm using mothers with completed and complete observation plus missed data for hemorrhage variable, Table S4: Sensitivity analysis of adjusted regression model for early term infants using mothers with completed and complete observation plus missed data for hemorrhage variable.
