# Supplementary material for: Factors Associated with Increased Risk of Early Severe Neonatal Morbidity in Late Preterm and Early Term Infants
Source: J Clin Med. 2021 Mar 23;10(6):1319. doi: 10.3390/jcm10061319 (PMC8004864; doi:10.3390/jcm10061319)
Supplement: Supplementary file 1 [file jcm-10-01319-s001.pdf]

Journal of Clinical Medicine

Article

Supplementary Material: Factors Associated with Increased Risk of Early Severe Neonatal Morbidity in Late Preterm and Early Term Infants

Tesfaye S. Mengistu, Veronika Schreiber, Christopher Flatley, Jane Fox and Sailesh Kumar

Table S1. Multivariable association of risk factors and early severe neonatal morbidity by gestational age category.

Risk Factors	Early SNM in Late Preterm (n = 172)	p-Value	Early SNM in Early Term (n = 182)	p-Value
	Adjusted OR (95%CI) ¥		Adjusted OR (95%CI) §	
Nulliparous	0.51 (0.35–0.75)	0.001	-	-
Maternal diabetes status				
No diabetes	Reference		Reference	
Pre-existing diabetes	3.30 (1.65–6.60)	0.001	3.25 (1.82–5.80)	<0.001
Gestational diabetes	0.85 (0.51–1.41)	0.523	1.59 (1.13–2.23)	0.007
Antepartum hemorrhage	-	-	1.91 (1.09–3.36)	0.024
Method of birth				
Spontaneous vaginal birth	Reference		Reference	
Instrumental birth	2.29 (1.03–5.11)	0.043	3.49 (2.18–5.60)	<0.001
Emergency CS for FTP	1.89 (0.61–5.82)	0.270	2.45 (1.22–4.93)	0.012
Emergency CS for NRFS	3.85 (1.82–8.13)	<0.001	2.82 (1.41–5.66)	0.003
Emergency CS Other	2.61 (1.61–4.22)	<0.001	3.11 (1.90–5.09)	<0.001
Elective CS	1.36 (0.78–2.36)	0.279	1.69 (1.09–2.62)	0.020
Birth weight (g)	-	-	1.00 (1.00–1.00)	0.029
Infant’s sex (Female vs male)	0.55 (0.38–0.80)	0.002	0.61 (0.45–0.84)	0.002

CS—caesarean section; FTP—failure to progress; NRFS—none-reassuring foetal status; ¥ Adjusted for: maternal age, ethnicity, gestational age at birth in weeks; § Adjusted for maternal age, ethnicity, maternal BMI, SEIFA Score and gestational age at birth in weeks.

Sensitivity analyses results

Table S2. Sensitivity analysis: Outcome and risk factors between cohorts with antepartum haemorrhage complete observation and no complete data.

Variable	Complete Data for Haemorrhage (n = 6243)	Missed Data for Haemorrhage (n = 12,636)	p-Value
Composite early SNM			<0.001
No	94.3% (5889/6243)	96.3% (12,167/12,636)	
Yes	5.7% (354/6243)	3.7% (469/12,636)	
Maternal age at birth (mean, sd)	32.7 (5.3)	32.5 (5.1)	0.025
Maternal ethnicity			<0.001
Caucasian	58.8% (3670/6243)	61.0% (7702/12,636)	
ASTI	4.0% (250/6243)	1.5% (195/12,636)	
Asian	24.7% (1542/6243)	25.4% (3204/12,636)	
Other	12.5% (781/6243)	12.1% (1535/12,636)	
SEIFA Score (median, IQR)	1037 (999–1073)	1040 (1001–1076)	<0.001

Household smoking status			0.11
Non-smoking household	81.8% (5108/6243)	82.4% (10,414/12,636)	
Mum smokes	10.5% (653/6243)	9.5% (1205/12,636)	
Partner smokes	7.7% (482/6243)	8.0% (1017/12,636)	
Illicit drug use during pregnancy			0.020
No	94.1% (5874/6243)	94.9% (11,992/12,636)	
Yes	5.9% (369/6243)	5.1% (644/12,636)	
Parity			<0.001
Multipara	55.5% (3466/6243)	58.0% (7334/12,636)	
Nullipara	44.5% (2777/6243)	42.0% (5302/12,636)	
Maternal BIM (kg/m^2) (mean, sd)	24.17 (21.2–28.91)	22.98 (20.57–26.61)	<0.001
Maternal diabetes status			<0.001
No diabetes	62.0% (3868/6243)	93.4% (11,807/12,636)	
Pre-existing diabetes	3.6% (222/6243)	1.1% (143/12,636)	
Gestational diabetes	34.5% (2153/6243)	5.4% (686/12,636)	
Hypertension			<0.001
No hypertension	87.8% (5482/6243)	94.4% (11,934/12,636)	
Essential/gestational hypertension	7.6% (474/6243)	3.8% (474/12,636)	
Pre-eclampsia/ eclampsia/ HELLP syndrome	4.6% (287/6243)	1.8% (228/12,636)	
Chorioamnionitis			0.003
No	99.0% (6179/6243)	99.4% (12,557/12,636)	
Yes	1.0% (64/6243)	0.6% (79/12,636)	
Assisted Reproduction			<0.001
No	86.9% (5427/6243)	89.3% (11,290/12,636)	
Yes	13.1% (816/6243)	10.7% (1346/12,636)	
Induction of labour			<0.001
No	60.7% (3790/6243)	67.9% (8586/12,636)	
Yes	39.3% (2453/6243)	32.1% (4050/12,636)	
Method of Birth			<0.001
Spontaneous vaginal birth	40.3% (2515/6243)	43.2% (5465/12,636)	
Instrumental birth	10.1% (633/6243)	10.7% (1346/12,636)	
Emergency CS for FTP	3.8% (237/6243)	3.1% (386/12,636)	
Emergency CS for NRFS	3.7% (233/6243)	3.2% (408/12,636)	
Emergency CS Other	12.7% (791/6243)	11.1% (1403/12,636)	
Elective CS	29.4% (1834/6243)	28.7% (3628/12,636)	
Infant's birth weight	3147.8306 (505.18002)	3180.2712 (480.64175)	<0.001
Fetus' sex			0.88
Male	52.7% (3291/6243)	52.8% (6676/12,636)	
Female	47.3% (2952/6243)	47.2% (5960/12,636)	

SNM—severe neonatal morbidity; sd—standard deviation, IQR—inter quartile range; BMI—body mass index; HELLP—haemolysis, elevated liver enzymes, low platelet count; IOL—induction of labour; CS—caesarean section; FTP—failure to progress; NRFS—none-reassuring fetal status.

Table S3. Sensitivity analysis of adjusted regression model for late preterm using mothers with completed and complete observation plus missed data for hemorrhage variable.

**Variable**	**Late Preterm Cohort ‡**			
	**Complete Data for Haemorrhage**		**With Missing Data for Haemorrhage**	
	**Adjusted OR (95% CI)**	***p*-Value**	**Adjusted OR (95% CI)**	***p*-Value**
Nullipara	0.51 (0.35–0.75)	0.001	0.57 (0.44–0.74)	<0.001
Maternal diabetes status				

No diabetes	Reference		Reference	
Pre-existing diabetes	3.30 (1.65–6.60)	0.001	3.24 (1.89–5.56)	<0.001
Gestational diabetes	0.85 (0.51–1.41)	0.523	0.96 (0.62–1.48)	0.84
Method of birth				
Spontaneous vaginal birth	Reference		Reference	
Instrumental birth	2.29 (1.03–5.11)	0.04	1.89 (1.13–3.17)	0.02
Emergency CS for FTP	1.89 (0.61–5.82)	0.270	1.56 (0.66–3.69)	0.32
Emergency CS for NRFS	3.85 (1.82–8.13)	<0.001	2.28 (1.35–3.84)	0.002
Emergency CS Other	2.61 (1.61–4.22)	<0.001	2.33 (1.70–3.19)	0.001
Elective CS	1.36 (0.78–2.36)	0.279	1.55 (1.06–2.25)	0.02
Infants sex (female vs male)	0.55 (0.38–0.80)	0.002	0.58 (0.45–0.74)	<0.001

CS—caesarean section; FTP—failure to progress; NRFS—none-reassuring fetal status; ‡ Adjusted for: maternal age, ethnicity, gestational age at birth in weeks.

Table S4. Sensitivity analysis of adjusted regression model for early term infants using mothers with completed and complete observation plus missed data for hemorrhage variable.

Variable	Early term cohort #			
	Complete Data for Haemorrhage		With Missing Data for Haemorrhage	
	Adjusted OR (95% CI)	*p*-Value	Adjusted OR (95% CI)	*p*-Value
Maternal diabetes Status				
No diabetes	Reference		Reference	
Pre-existing diabetes	3.25 (1.82–5.80)	<0.001	2.92 (1.87–4.57)	<0.001
Gestational diabetes	1.59 (1.13–2.23)	0.007	1.56 (1.22–1.99)	<0.001
Method of Birth				
Spontaneous vaginal birth	Reference		Reference	
Instrumental Birth	3.49 (2.18–5.60)	<0.001	3.93 (2.93–5.27)	<0.001
Emergency CS for FTP	2.45 (1.22–4.93)	0.012	3.00 (1.92–4.71)	<0.001
Emergency CS for NRFS	2.82 (1.41–5.66)	0.003	4.44 (2.94–6.71)	<0.001
Emergency CS Other	3.11 (1.90–5.09)	<0.001	3.26 (2.39–4.46)	<0.001
Elective CS	1.69 (1.09–2.62)	0.02	1.92 (1.45–2.54)	<0.001
Infants sex (female vs male)	0.61 (0.45–0.84)	0.002	0.64 (0.53–0.78)	<0.001
Infant's birth weight (continuous)	1.00 (1.00–1.00)	0.029	1.00 (1.00–1.00)	0.69

Adjusted for maternal age, ethnicity, SEIFA score and gestational age at birth in weeks.
